# Determining the effect of COVID-19 on antibiotic use in long-term care facilities across Tennessee

**DOI:** 10.1017/ash.2022.93

**Published:** 2022-05-16

**Authors:** Cullen Adre, Youssoufou Ouedraogo, Christopher Evans, Christopher Wilson

## Abstract

**Background:** Nationally, a decrease in total antibiotic use in nursing homes during the COVID-19 pandemic was observed with an increase in select agents used for respiratory infections. Currently there is minimal data on antibiotic use in long-term care facilities (LTCFs) in Tennessee. To address this issue, the Tennessee Department of Health (TDH) developed a monthly point-prevalence survey of antibiotic use. Utilizing this tool, we sought to determine the effect the pandemic had on antibiotic use in Tennessee LTCFs. **Method:** We developed a REDCap questionnaire to collect information on selected antibiotics administered in Tennessee LTCFs. Antibiotic use percentage was determined by dividing the number of residents who received an antibiotic on the day of survey by facilities’ average censuses. Data were divided into a prepandemic period (January 2019–February 2020) and a period during the pandemic (March 2020–December 2021). Antibiotic prescriptions were grouped into 4 classes according to their most common uses: *Clostridium difficile* infections, urinary tract infections, skin and soft-tissue infections (SSTIs), and respiratory infections. Average percentage of residents on antibiotics were compared between study periods. **Results:** In total, 37 facilities participated in the survey during the prepandemic period and 32 facilities participated during the pandemic period; 14 participated during both periods. The average percentage of residents on antimicrobials before the pandemic was 16.3%, which decreased to 11.5% during the pandemic period (*P* = .04). During the prepandemic period, 40.2% of antibiotics prescribed were in the common for SSTI category and 38.3% were in the common for respiratory infections category (*P* = .01); during the pandemic period, 64.3% of antibiotics prescribed were in the common for SSTI category and 45.8% were in the common for respiratory infections category (*P* = .01). The 3 most prescribed antibiotics in the prepandemic period were amoxicillin (148 prescriptions), doxycycline (140 prescriptions), and levofloxacin (135 prescriptions). The 3 most prescribed antibiotics during the pandemic were doxycycline (141 prescriptions), levofloxacin (125 prescriptions), and trimethoprim–sulfamethoxazole (115 prescriptions). **Conclusions:** Survey results revealed that antibiotic prescriptions commonly used for respiratory infections increased 7.5% during the pandemic study period. Additionally, the average percentage of residents on antimicrobials fell 4.8% during this period. Both statistics reflect what has been seen nationally with a decrease in antibiotic use with an increase in respiratory antibiotics. This could be due to multiple factors including decreased reporting, a change in healthcare delivery during the pandemic, and facilities seeing an increase of respiratory tract infections. These data will be used to guide future TDH antibiotic stewardship efforts in the long-term care setting.

**Funding:** None

**Disclosures:** None

